# Construction of High-Density Genetic Map and Identification of QTLs Associated with Seed Vigor after Exposure to Artificial Aging Conditions in Sweet Corn Using SLAF-seq

**DOI:** 10.3390/genes11010037

**Published:** 2019-12-28

**Authors:** Xiaming Wu, Faqiang Feng, Yuzhong Zhu, Fugui Xie, Jing Yang, Jie Gong, Yu Liu, Wei Zhu, Tianle Gao, Danyi Chen, Xiaoqin Li, Jun Huang

**Affiliations:** 1 The Key Laboratory of Plant Molecular Breeding of Guangdong Province, College of Agriculture, South China Agricultural University, Guangzhou 510642, China; XiamingW@stu.scau.edu.cn (X.W.); fengfq@scau.edu.cn (F.F.); zhuyuzhong001@163.com (Y.Z.); xiefugui@stu.scau.edu.cn (F.X.); cherry_18589268923@163.com (J.G.); YuLiu2019@126.com (Y.L.); zhuwei@stu.scau.edu.cn (W.Z.); gtl@stu.scau.edu.cn (T.G.); 15813323952@163.com (D.C.); xiaoqinli2000@126.com (X.L.); 2 National Engineering Research Center of Plant Space Breeding, South China Agricultural University, Guangzhou 510642, China; 15989091552@163.com

**Keywords:** SLAF, QTL, seed vigor, artificial aging

## Abstract

Seed vigor is a key factor that determines the quality of seeds, which is of great significance for agricultural production, with the potential to promote growth and productivity. However, the underlying molecular mechanisms and genetic basis for seed vigor remain unknown. High-density genetic linkage mapping is an effective method for genomic study and quantitative trait loci (QTL) mapping. In this study, a high-density genetic map was constructed from a 148 BC_4_F_3_ population cross between ‘M03’ and ‘M08’ strains based on specific-locus amplified fragment (SLAF) sequencing. The constructed high-density genetic linkage map (HDGM) included 3876 SNP markers on ten chromosomes covering 2413.25 cM in length, with a mean distance between markers of 0.62 cM. QTL analysis was performed on four sweet corn germination traits that are related to seed vigor under artificial aging conditions. A total of 18 QTLs were identified in two seasons. Interestingly, a stable QTL was detected in two seasons on chromosome 10—termed *qGR10*—within an interval of 1.37 Mb. Within this interval, combined with gene annotation, we found four candidate genes (*GRMZM2G074309*, *GRMZM2G117319*, *GRMZM2G465812*, and *GRMZM2G343519*) which may be related to seed vigor after artificial aging.

## 1. Introduction

Sweet corn (*Zea mays* L. saccharata Sturt) is a maize-derived vegetable crop with one or more recessive endosperm mutations that reduce the synthesis of starch and increase the accumulation of sugars or other short chain polysaccharides. In recent years, the land area planted with sweet corn in China has expanded rapidly. The total area was approximately 323,000 hm^2^ in 2012. Thus, it has become an important superior and efficient economic crop in the southern regions of China such as Guangdong, Guangxi, Zhejiang, Yunnan, etc. [1]. Consequently, the demand for sweet corn which has gradually become an indispensable green health food has increased as improvements in quality of life have been achieved. However, one of the most troublesome problems is low seed vigor in sweetcorn seeds. The low germination rate and uneven emergence of seedlings from sweet corn seeds in fields has led to low harvest yields and high prices for sweet corn seeds. Furthermore, sweet corn seeds are extremely difficult to preserve with seed vigor decreasing dramatically over time. For the above reasons, the production of sweetcorn in China is unable to satisfy the demand.

Seed vigor is the most important characteristic of seed quality. According to different methods of identification, the principal evaluation indices include germination potential, germination rate, seedling length, fresh weight, dry weight, low temperature germination capability, storage resistance, and aging resistance. Many studies have confirmed that seed vigor is highly heritable, with genetic factors largely determining the vigor of seeds, so there are hereditary differences in seed vigor among different crop species and varieties. At present, genetic studies on seed vigor have been conducted in rice [2,3,4], wheat [5,6], barley [7], and maize. Styer and Cantliffe (1984) used a near isogenic line to analyze the seed vigor of four types of maize mutants (*brittle-1*, *shrunken-2*, *sugar* and *normal*). They found that the *shrunken-2* mutant had the least quantity of seed starch, with the embryo accounting for 25% of total dry weight of the seed, in which the seed vigor and germination rates were lower in adverse conditions [8]. TeKrony and Hunter (1995) used maize inbred lines (B73 and Mo17) single and double cross hybrids to study seed vigor in maize at different stages of maturity. It was found that the seed vigor of single- and double- cross progeny reached their maximum at black layer stage 4. For inbred maize, the maximum seed vigor appeared slightly later [9]. Li et al. (2007) found that mutations in lipid oxidase isoenzyme genes *LOX-1* and *LOX-2* decreased maize seed vigor [10].

Over the past decade, genetic maps have been constructed using traditional molecular markers which commonly have a low marker density in general. These previously constructed genetic maps did not cover the whole genome [11]. Nowadays, next-generation sequencing (NGS) technology has developed rapidly, providing an effective method of detecting a large number of SNP markers across the entire genome. Specific-length amplified fragment sequencing (SLAF-seq), is a novel simplified genome sequencing technology based on high-throughput sequencing, which was first developed by Sun et al. [12]. Using this method, an SLAF library has been constructed based on the SLAF pilot experiments in accordance with the pre-designed schemes, and specific restriction endonucleases were used to digest the genome. After enzyme digestion, the specific lengths of DNA fragments (i.e., “SLAF tag”) were screened, and the polymorphic SLAF tags were obtained by sequence similarity analysis, allowing for the development of specific molecular markers. SLAF-seq has allowed high-density genetic maps for several crops to be constructed, such as peanut [13], upland cotton [14] and sunflower [15]. Therefore, the construction of high-density genetic maps of sweet corn based on SLAF markers is possible.

Seed vigor is a complex comprehensive quantitative trait affected by multiple factors [12], making the genetic analysis of seed vigor difficult as a result. With the rapid development of high-throughput sequencing technologies and genome graphing techniques, quantitative trait loci (QTL) analysis has provided a powerful tool with which to study the inheritance of seed vigor. Compared with a bulked segregant analysis (BSA) approach, both the sensitivity and accuracy of QTL analyses are higher and can be used for other traits; thus, scientific research of a greater depth can be conducted. QTL analyses with traits related to seed vigor have been utilized in rice [16], barley [17], wheat [18], oilseed rape [19] and *Arabidopsis thaliana* [20], using tests of artificial aging. To our knowledge, only two genes regulating seed vigor obtained by map-based cloning have been reported. Fujino et al. (2008) constructed a backcross inbred line using two *japonica* rice materials (Hayamasari and Livorno, Italy) as parents. A major QTL was located on chromosome 3 that regulates the ability of rice to germinate at low temperatures termed *qLTG3-1*. *qLTG3-1* was found to be encoded by a protein with an unknown function though map-based cloning. The expression of the gene in the aleurone layer of the seed coat and the epiderm covering the coleoptile may increase the potential for the germination and vigor of seeds at low temperatures through the regulation of cell vacuolation in these tissues [21]. Subsequently, the target genes of *qLTG3-1* were identified by genome-wide analysis. A total of 4587 differentially expressed genes were detected and 29 target genes identified [22]. The function of these genes indicates that cell autoregulation might lead to a wide range of metabolic changes and interactions between different signaling pathways. *qLTG3-1* is able to upregulate the expression of defense response genes, indicating that the gene is located upstream of its regulation. The following year, Fujino and Skiguchi (2011) studied *qLTG3-1* alleles and their distribution in 62 Asian rice landraces. A non-synonymous alternative SNP and three insertion-deletion polymorphic loci were detected in the coding region, allowing functional markers to be developed [23]. Nguyen et al. (2012) compared six recombinant inbred line (RIL) populations using different measures of vigor to reveal the genetic regulators of seed longevity and its association with seed dormancy in *Arabidopsis* which revealed a candidate gene (*DOG1*). It also described an important negative correlation between seed longevity and seed dormancy found in this study [24]. To our knowledge, there are only a few reports of linkage analysis using artificial aging to map QTL related to maize seed vigor [25,26]. Although the studies above have mapped the major QTLs, no genes relating to seed vigor have yet been cloned in maize. Therefore, in this study, BC_4_F_3_ populations were grown in artificial aging conditions to identify the QTLs for traits related to seed vigor to provide the theoretical basis and support for the cultivation of high-vigor varieties of sweet corn.

## 2. Materials and Methods

### 2.1. Construction of Plant Population

The inbred sweet corn line M03 (recurrent parent) was crossed with another inbred sweet corn line M08 (donor parent) to create F_1_ crosses during autumn 2010 in the Zengcheng Experimental Base of South China Agricultural University. Following 4 backcrosses and 3 self-crosses the BC_4_F_3_ mapping population was created which included 148 lines, from which a high-density genetic map (HDGM) was constructed. The genetic background recovery rates were calculated as S/T, where S is the number of markers consistent with the recurrent parents in an individual, T is the total number of markers in an individual. Finally, there were only 8 lines whose genetic background recovery rates were less than 90%. The recovery rates of all other lines were higher than 90%, the highest rate being 99.99%, with a mean recovery rate of 95.91%. All plant materials used in this study were collected by Prof. Xiaoqin Li (College of Agriculture, South China Agricultural University, Guangzhou, China).

A total of 148 lines from two parents were grown in the Zengcheng Experimental Base of South China Agricultural University (at a longitude of approximately 113° East and latitude of approximately 23° North) in spring and autumn 2017. Ten plants of each line or parent were grown with 2 replicates. The length of each row was 3 m with a row spacing of 70 cm. Plant spacing was 25 cm, at a density of 57000 per hm^2^. Crop management, and disease and insect pest control were performed as recommended locally. Prior to the plants forming blossoms, the stamens and pistils of the sweet corn were bagged and self-pollinated to obtain homozygous seeds. All seeds were harvested 50 days after pollination and stored in a seed storage room until required for additional study.

### 2.2. Artificial Aging Treatment

Prior to performing the aging procedure, the thermostatic moisture regulator was operated for 6 h to ensure that the temperature and humidity were balanced. Samples of seeds from the 148 BC_4_F_3_ lines and their parent lines were incubated in the regulated environment at 41 °C and 99% relative humidity for 3 days. Untreated samples were used as controls. After aging, the seeds were removed and air-dried for 3 days, until the seed humidity was less than 14%.

### 2.3. Experimental Design and Phenotypic Evaluation

Three repeats of seed germination were conducted in a growth chamber at 25 °C using a randomized complete block design. The cultivation medium consisted of fine sand with particles with a diameter of no more than 0.2 mm. It was rinsed with clean water to remove dirt and toxic substances then heated to 120 °C for 2 h in a high-handed sterilization pan. Forty seeds from three different plants of the parental lines and each of the BC_4_F_3_ population were soaked in 0.1% sodium hypochlorite solution for 15 min, then rinsed three times with sterile distilled water. The seeds were placed on the surface of a 3 cm depth of sand in each well and then covered with an additional 2 cm of sand. All wells were placed in an incubator at 25 °C, 65% relative humidity for 7 d with a 14/10 h (day/night) photoperiod. The germinated seeds were observed each day for 7 day, after which almost all the seeds had germinated. Four traits associated with seed vigor were identified. Germination potential (GP) was the percentage of seeds that had germinated after 4 days. Germination rate (GR) was defined as the percentage of seeds that had germinated after 7 days. The germination index (GI) was modified according to the method of Cao et al. (2008): *GI* = Σ(G_t_/t) [27], where *G_t_* was the number of germinated seeds on Day *t*. The vigor index (VI) was calculated by: *VI* = *ΣGI* × *S*, where *S* was the weight (g) of a 7 day normal seedling.

The phenotypic data were analyzed using SPSS version 19 (SPSS Inc., Chicago, IL, USA). These variance components of the genotype and environment were estimated in a linear mixed model (Proc Mixed) in SAS version 9.4 (The SAS Institute, Cary, NC, USA). The broad sense heritability (h^2^) was estimated using the following formula: h^2^ = σg^2^/(σg^2^ + σe^2^/r), where σg^2^ is the genetic variance, σe^2^ is the residual error, and r is the number of replications.

### 2.4. SLAF Library Construction

Genomic DNA was extracted from the two parents and the BC_4_F_3_ population using a cetyl trimethylammonium bromide (CTAB) protocol, as previously described [28]. The restriction enzyme Hpy166II was utilized to digest the genomic DNA of all the BC_3_F_4_ population and two parents. The SLAF-seq concept has been described in detail previously [12]. Finally, 414–444 bp DNA fragments were used for paired-end sequencing on an Illumina HiSeq 2500 system (Illumina, Inc., San Diego, CA, USA).

### 2.5. Grouping and Genotyping of Sequence Data

The procedure described by Sun et al. [12] and Zhang et al. [29] was used for the identification and genotyping of SLAF markers. Briefly, remaining reads were mapped to the reference genome using BWA software after filtering out low-quality reads [30]. Sequences that had greater than 95% similarity were defined as identical SLAFs. Only SLAF markers that were consistent with parents and offspring were genotyped.

In order to create a high-quality genetic map, low-quality SLAFs were filtered out. The protocol for data filtering has been described previously [29]. One SLAF locus could harbor no more than four SLAF tags. Groups with more than four tags were considered repetitive SLAFs and discarded. Polymorphic SLAFs containing 2–4 tags were regarded as potential markers. These polymorphic SLAFs were used to construct the high-density genetic maps for sweet corn.

### 2.6. Linkage Map Construction

A linkage map was constructed in accordance with the procedure described by [29]. SLAF markers were divided into ten linkage groups (LGs) based on their location. Genotyping errors and the deletion of the next-generation sequencing data reduced the quality of the high-density genetic maps. SLAF markers were ordered in terms of high map strategy and smoothing strategy. Genotyping errors in the linkage groups were corrected [31,32]. The imputation of missing genotypes was performed using the K-nearest neighbour algorithm based on the two parents and the BC_4_F_3_ population [33]. Skewed markers were added to the linkage map through the application of a multipoint method of maximum likelihood [34]. SLAFs with an MLOD value of less than 5 were filtered out, with calculation of MLOD values between two adjacent markers [35]. The Kosambi mapping function was utilized to estimate the genetic distance between two adjacent markers [36]. SNPs were coded according to the genotype of the parents. The genotype of the offspring was ascertained according to the relationship between offspring and their genotype. For example, if the parent genotypes of a marker were aa and bb, the offspring genotype ab indicates that the parents of these two SNP loci were homozygous in this SNP position. All polymorphic SLAFs were divided into eight segregation patterns (ab × cd, ef × eg, hk × hk, lm × ll, nn × np, aa × bb, ab × cc, and cc × ab) based on the genotype encoding rule. Thus, only plants with an aa × bb segregation pattern were used to construct the high-density genetic linkage map.

### 2.7. QTL Analysis

QTL mapping was conducted using a composite interval mapping (CIM) method using QTLNetwork v2.1 software [37]. To determine the LOD threshold, each trait in each environment was tested separately by permutation tests 1000 times (α = 0.05). By default, a 10 cM window was used, with the background marker set to 5. The background was controlled using a forward-reverse stepwise regression method with a step size of 1 cM. Moreover, the QTL action mode was determined according to the criteria proposed by Stuber [38]. The designation of each QTL was: q, plus an abbreviation defining its traits and the chromosome on which the QTL was located, multiple QTLs on the same chromosome designated numerically. QTL designations in this paper are expressed in italics. Thus, *qGR1-3* indicates the third QTL on chromosome 1 detected in the controlled germination rate population.

## 3. Results

### 3.1. Analysis of SLAF-seq Data

A total of 57.06 Gb raw data were obtained based on SLAF-seq which contained 228.26 M paired-end reads, the length of which was 100 base pairs (BP). Among them, guanine–cytosine (GC) content was 45.15%, and 86.39% of the bases were of high quality with Q30. A total of 12,759,218 reads were detected in the male parent and 12,276,986 in the female. The mean number of reads for the BC_4_F_3_ population was 1,370,055. Subsequently, 163,229 SLAFs were detected, of which there were 135,956 and 90,506 SLAFs in the male and female parents, respectively. Mean sequencing depths were 36.74-fold and 47.57-fold, for the male and female parent, respectively. In the BC_4_F_3_ population, the mean number of SLAFs was 98,023, with a mean sequencing depth of 5.47-fold for each offspring (Table 1).

Among the 163,229 SLAFs, 31,486 markers were identified as polymorphic in the whole BC_4_F_3_ population, a polymorphism rate of 19.20%. The majority of polymorphic SLAF markers were mapped onto chromosome 1 with 3972 SLAFs, with chromosome 10 having the least number of polymorphic SLAF markers with only 1997 (Table S1). Finally, 3876 SNPs were used to construct the linkage map after filtering out low-quality SLAFs.

### 3.2. High-Density Genetic Map Construction

**Figure 1:**
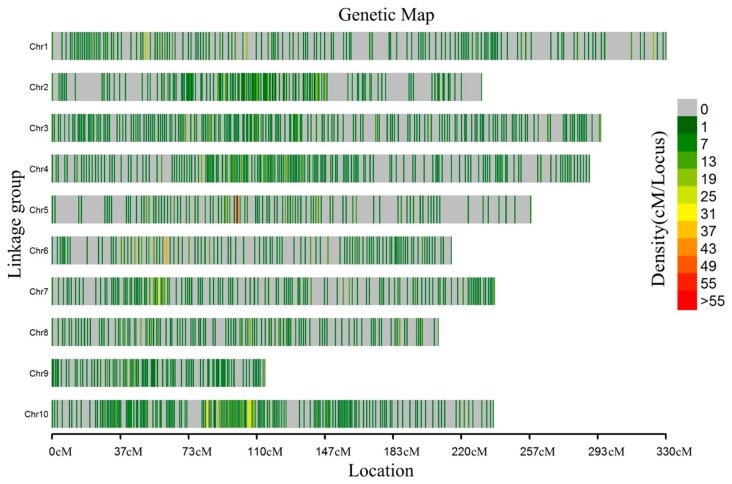
Genetic map of the 148 BC_3_F_4_ population. Colorful stripes indicate the distribution of markers on the 10 chromosomes and different colors indicated different densities.

There were 3876 SNP markers mapped on the genetic map after linkage analysis (Figure 1). The length of the genetic map was 2413.25 cM with a mean inter-marker distance of 0.62 cM. Chromosome 10 contained the largest number of markers with 518, having a length of 237.2264 cM and mean distance of 0.46 cM between markers. Chromosome 9 had the smallest number, with only 179 markers. The length of this chromosome was 114.32 cM with a mean distance of 0.64 cM. “Gap ≤ 5” reflected the degree of linkage between the markers, ranging from 90.86% to 99.26%, with a mean value of 96.44%. The largest gap on this map was located on LG05 at 15.28 cM, followed by 14.20 cM on LG01 (Table 2).

### 3.3. Quality and Accuracy of the Map

**Figure 2:**
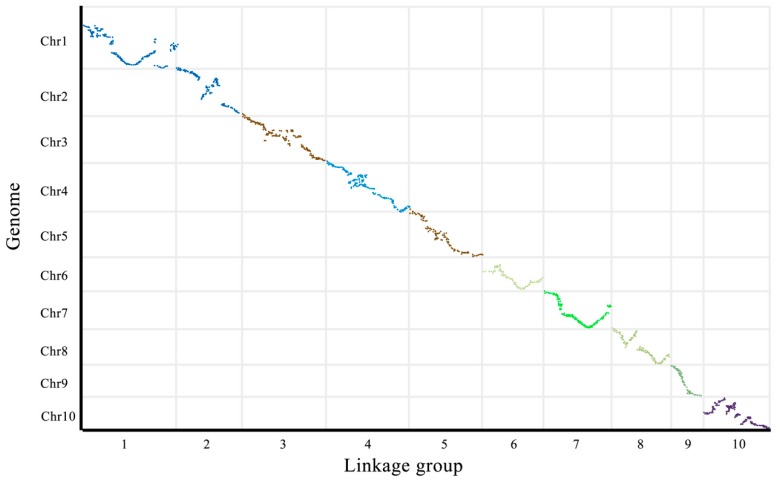
Scattered colinear maps of genetic maps and genomes. The *X*-axis is the genetic distance of each linkage group and the *Y*-axis is the physical length of each linkage group. Scatter points represent marker collinearity between genome and genetic map. The more diagonal the marker, the better the collinearity between the transferred map and genome.

A collinearity analysis of the genetic map and genome was performed to assess the quality and accuracy of the genetic map. A Spearman correlation coefficient for each linkage group was calculated. The closer the Spearman coefficient was to 1, the better the collinearity between the genetic map and genome. The order of the majority of markers on each linkage group in this study was consistent with the genome, indicating that collinearity was good and that the accuracy of calculation of the genetic recombination rate was high (Figure 2; Table S2).

### 3.4. Phenotypic Analysis

**Figure 3:**
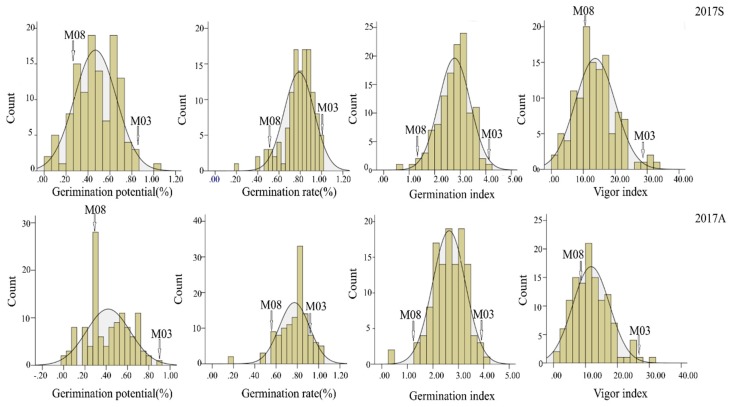
Frequency distribution of traits related to seed vigor under artificial aging treatments for the 148 BC_4_F_3_ population and two parents.

There were significant differences in GP, GR, GI, and VI between M08 and M03 parental lines under artificial aging condition. The M03 showed good performance of seed vigor, with higher GP, GR, GR, and VI. The BC_4_F_3_ also showed statistically significant differences in the four traits (*p* < 0.01). The GP of the individual BC_4_F_3_ population ranged from 0.00 to 1, GR ranged from 0.16 to 1.0, GI from 0.46 to 4.24, and VI from 0.19 to 32.41. The skew and kurtosis of the traits ranged from −1.28 to 0.58 and −0.80 to 3.63, respectively. The coefficients of variation ranged from 17.81 to 49.20, and the broad sense heritability (h^2^) ranged from 0.64 to 0.75 (Table 3). The frequency distributions of germination traits for the BC_4_F_3_ population are displayed in Figure 3. Germination trait data suggests that the segregation of those traits fits a normal or skewed normal distribution, indicating that seed vigor was a typical quantitative trait controlled by polygenes, and further analyzed for QTL detection.

**Figure 4:**
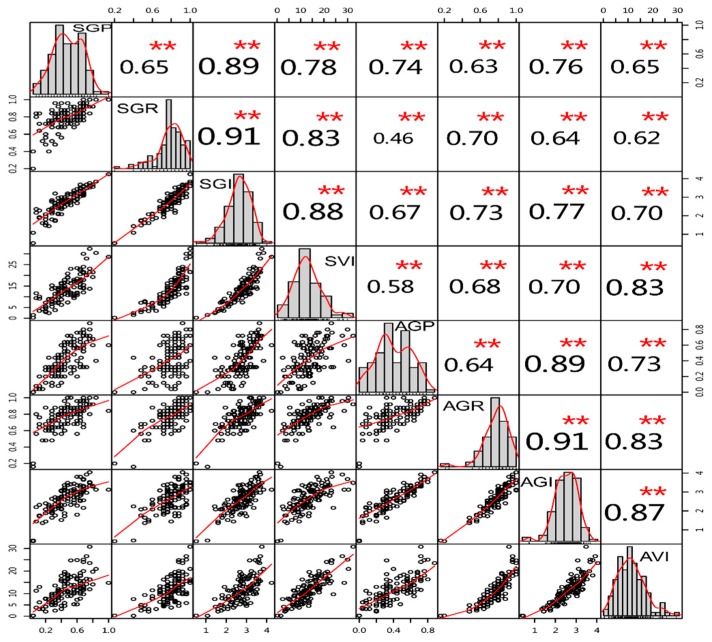
Correlation coefficients among the four traits at the germination stages after artificial aging treatments in the BC_4_F_3_ population across two seasons. The upper panel contains the correlation coefficient correlations, where ** represents significance at 0.01. In the lower panel, scatter plots represent the degree of dispersion between two traits. The diagonal represents the frequency distribution of the four traits. SGP: germination potential in spring 2017; SGR: germination rate in spring 2017; SGI: germination index in spring 2017; SVI: vigor index in spring 2017; AGP: germination potential in autumn 2017; AGR: germination rate in autumn 2017; SGI: germination index in autumn 2017; SVI: vigor index in autumn 2017.

Correlation analyses between changes in the four germination traits are helpful in identifying the link between them relating to seed vigor. Overall, the correlation coefficients of the four germination traits were found to be statistically significant (*p* < 0.01) and positively correlated with each other, ranging from *r* = 0.46 to 0.91 (Figure 4).

### 3.5. QTL Analysis of Traits Associated with Seed Vigor in the BC_4_F_3_ Population

**Figure 5:**
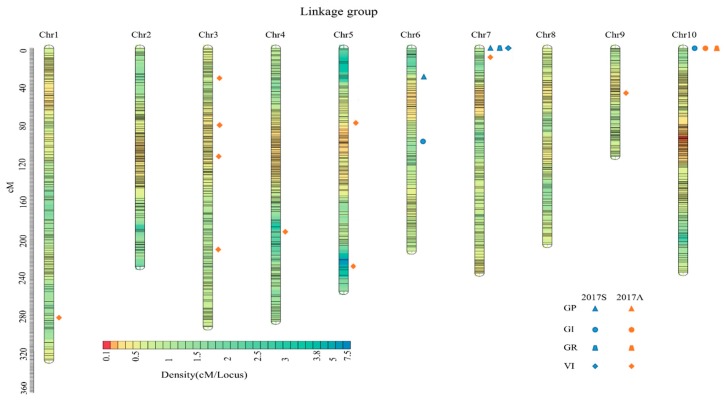
Distribution of the 18 QTLs identified in this study related to seed vigor under artificial aging condition across two seasons on the 10 chromosomes. (2017S: 2017 spring; 2017A: 2017 autumn).

18 QTLs for GP, GR, GI, and VI were mapped for the BC_4_F_3_ populations germinated under artificial aging conditions across two seasons in 2017 (six for the spring, 12 for the autumn) (Table 4; Figure 5). These QTLs are mapped to chromosomes 1, 3, 4, 5, 6, 7, 9, and 10 in sweetcorn. The phenotypic variation explained by individual QTLs for four germination traits ranges from 0.47% to 13.18% at a LOD of 2.05–5.85. Seven QTLs (*qGP6*, *qVI1*, *qVI3-3*, *qVI4*, *qVI5-1*, *qVI5-2*, and *qVI9*) had a positive additive effect, indicating that alleles are from the male parent (M03), while the negative effects of the remaining QTLs are derived mainly from the female parent (M08).

Two QTLs associated with GP and GR were found on chromosomes 6 and 7 and chromosomes 6 and 10, the phenotypic variance explained by a single QTL ranging from 0.617% to 1.689% and 2.379% to 13.138%, respectively. Two QTLs (*qGI7* and *qGI10*) were responsible for the germination index located on chromosomes 7 and 10, respectively, explaining a total of 9.559% of the phenotypic variance. Eleven QTLs controlling vigor index were identified on chromosomes 1, 3, 4, 5, 7, and 9, respectively, accounting for 20.014% of the phenotypic variance. *qGR10* and *qGI10* were in the same QTL region which could explain 8.070–13.138% of the phenotypic variation. *qGP7*, *qGI7*, and *qVI7* mapping to the same locus explained 0.617–1.489% of the phenotypic variation, which indicated that *qGR10* was the major stable locus interval (73,141,877–74,489,350) associated with sweetcorn seed vigor.

### 3.6. The Candidate Gene Analysis

**Figure 6:**
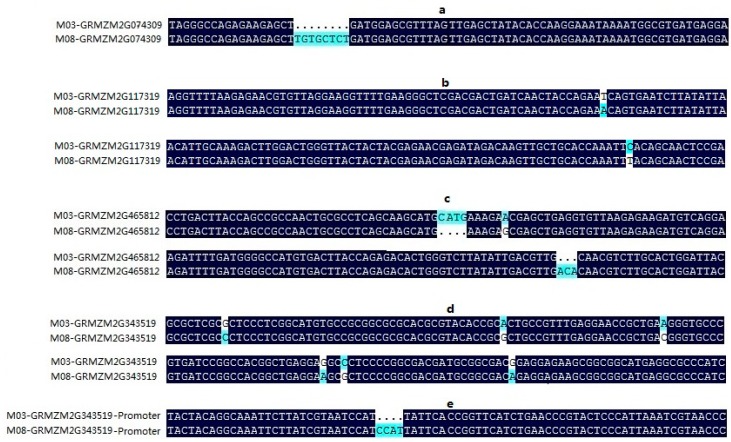
The promoter and coding sequence difference of four genes between M03 and M08 line. The shading of the alignment presents identical regions in dark black, different regions in light cyan. Dots denote gaps. (**a**–**d**) indicated the difference of *GRMZM2G074309*, *GRMZM2G117319*, *GRMZM2G465812*, and *GRMZM2G343519* in the CDS region between two parental lines, respectively, (**e**) was the difference of *GRMZM2G343519* in the promoter region between two parental lines).

A 1.37 Mb common QTL region *qGR10* of 73,141,877–74,489,350 on chromosome 10 was identified, which could explain 8.070% to 13.138% of phenotypic variation (Table 4). In total, twenty-five genes within the region of *qGR10* were found according to maize inbred line B73 reference genome Zea_mays_v3 (download address: ftp://ftp.ensembl genomes.org/pub/plants/release-24/fasta/zea_mays). Twenty-five candidate gene annotations are listed in Table S3. Combined with gene annotation, we found four candidate genes (*GRMZM2G074309*, *GRMZM2G117319*, *GRMZM2G465812*, and *GRMZM2G343519*) which may be related to seed vigor after artificial aging. Furthermore, the promoter and coding sequence (CDS) of those four genes of the M03 and M08 lines were sequenced. *GRMZM2G074309* in the M03 line was missing a TGTGCTCT sequence in the CDS region compared with the sequence from the M08 line. There were two SNPs in the CDS region of *GRMZM2G117319* between the M03 and M08 lines. *GRMZM2G465812* in the M03 line had inserted a CATG but was missing an ACA in the coding sequence compared with the M08 line. Though there were only several SNPs in the CDS, *GRMZM2G343519* had a copy of CCAT in the promoter of the M08 line compared with M03 (Figure 6). Variation in these three genes may lead to the differences in seed vigor between the two parents. This information will aid in the fine mapping of the qGR10 locus, with further experiments needed for the identification of functional genes and the discovery of the causes of the differences in seed vigor.

## 4. Discussion

Seed vigor plays an important role in agricultural production. Higher seed quality is required for the enhanced development of agricultural production. When seeds are stored, higher seed vigor provides greater resistance to various adversities. Therefore, it is helpful for the long-term preservation of the germplasm resource. It is of great significance in the production of seeds to understand the relationship between seed aging and seed vigor. For maize, seed vigor has had a profound effect on plant morphogenesis and germination [39,40]. Seed aging not only affects seed germination, seedling development, and the yield and quality of later-harvested seeds, but also the storage and utilization of germplasm resources. Artificial aging is a process in which seeds are placed under artificially controlled conditions, causing germination rate, germination potential and seed vigor to decline rapidly, which is an effective way to study the regulation mechanism of seed degradation. Few studies to date have reported on the molecular mechanisms of traits related to seed vigor under artificial aging treatments in maize. In this study, a subset of the BC_3_F_4_ population was used as the research object to evaluate four seed vigor traits after artificial aging prior to germination. In future breeding, seed vigor could be used as a breeding selection standard, to obtain high yields and high-quality hybrids.

### 4.1. Characteristics of the SLAF-seq Method

SLAF-seq is a novel high-throughput sequencing technique suitable for large-scale genotyping that has been developed over recent years. Traditional gene mapping methods are based on the cloning of important agronomic traits which require the isolation of a large number of individual genotypes in the population. This method is time-consuming, laborious and costly, now replaced by a new molecular method for simplified genome sequencing, SLAF-seq. Compared with AFLP, RAPD, RFLP, and SSR markers, SLAF-seq has the advantages of high marker development efficiency, low cost and large population capacity. The repeatability of SLAF-seq is higher than other sequencing techniques (such as RAD-seq, double digest RAD-seq or GBS), using the sequencing of paired-ends of sequence-specific restriction fragment lengths. The key step in the construction of high-density genetic maps is the selection of appropriate restriction endonucleases. The numerous stable and reliable molecular markers generated by next generation sequencing is a better representation of the genome, as appropriate restriction endonuclease(s) were applied to genome digestion.

In this study, total SLAF-seq data size was 57.06 Gb, of which 163,691 were high-quality SLAF tags, including 31,582 polymorphic SLAF tags. The markers obtained covered all sweetcorn chromosomes, and the number of polymorphic SLAF markers on each chromosome ranged from 1997 to 3972. The final high-density genetic map contained 3876 SNP markers. Therefore, SLAF-seq technology can be regarded as an economical and effective technique for the development of chromosome-specific molecular markers in sweetcorn.

### 4.2. QTL Mapping of Seed Vigor Related Traits Compared with Other Studies

Seed vigor is a complex, comprehensive quantitative trait influenced by two major factors: genetic background and environmental factors. With the rapid development of molecular biotechnology and bioinformatics, QTL mapping of seed vigor-related traits has made great progress, especially in rice [2,3,4], but few studies have been published on maize. Previous studies have shown that in a total of 168 F_2:4_ families—crossed by two cultivars of differing seed vigor used as a mapping population—forty QTLs were found to be linked to seed vigor traits with two major QTLs mapped to chromosomes 4 and 5 [41]. In addition, 16 QTLs associated with seed vigor were mapped on chromosomes 2, 3, 4, 5, 7, 8, 9, and 10. Using the maize recombinant inbred line (RIL) population 32, 40 and 45 days after pollination, four were detected across three maturation stages of seeds [42]. Han et al. (2014) used SNP markers to analyze the four seed vigor traits of two linked RIL maize populations during seed germination under four treatments [25]. Sixty-five QTLs were identified on chromosomes 1, 3, 4, 5, 6, 7, and 10 on an integrated genetic map using meta-analysis, of which sixty-one initially identified QTLs with a phenotypic variation value of R^2^ > 10% were integrated into 18 meta-QTLs. Finally, twenty-three candidate genes related to seed vigor traits coincided with 13 mQTLs. Wang et al. (2016) mapped 49 QTLs on chromosomes 1, 2, 3, 4, 5, 6, 7, 8, and 10 with a maize-immortalized F_2_ population and its corresponding RILs under aging conditions. QTLs with a contribution to phenotypic variation values of R^2^ > 10% were found, except for on chromosomes 8 and 9, but only *qGP5* was detected across the two populations [26].

According to the results of previous studies, this study analyzed QTLs related to the seed vigor of sweetcorn under artificial aging conditions. A total of 18 QTLs were detected, which were distributed across chromosomes 1, 3, 4, 5, 6, 7, 9, and 10. The aging germination traits mapped one major QTL (qGP10) on chromosome 10 with region positions of 0.000–1.400 cM. This is similar to the results of major QTL locations identified by Liu et al. [41] and Wang et al. [26]. Unlike Han et al. [23], Liu et al. [43] and Wang et al. [26] also found major QTLs on chromosome 10 at chromosomal regions 10.07 cM and 10.05–10.07 cM, respectively. The results indicate that chromosome 10 is an important chromosome that controls seed vigor. Seed vigor QTLs were detected on chromosomes 3, 4, 5, 7, and 10, which is consistent with the results of Liu et al. [42]. The seed vigor QTLs mapped to chromosomes 1, 3, 4, 5, 6, 7 and 10 were consistent with the results of Han et al. [25]. In addition, seed vigor QTLs were detected on chromosomes 1, 3, 4, 5, 6, 7, and 10, which were consistent with the results of Wang et al. [26]. The reason that these correlated traits are often mapped at different positions could be because the significance of many QTLs is just above the LOD threshold and small differences in the accuracy of the estimates cause QTLs to be significant for some traits, but not for others. Although the major QTL loci were identified in the studies mentioned above, no map-based cloning of maize seed-aging genes has been reported. The qGP10 detected in this experiment is a new major QTL, providing an important reference for the cloning of maize seed-aging genes.

### 4.3. Candidate Genes Analysis

Based on gene annotations, *GRMZM2G343519* is a glutaredoxin family protein. During seed aging, the products of alcohol fermentation, lipid peroxidation, and the Maillard reaction are released, resulting in the accumulation of toxic substances [43]. The sulfhydryl group of reduced glutathione can combine with various electrophilic and lipophilic substrates to form water-soluble products under the action of glutathione transferases (GSTs), thus preventing damage from endogenous toxic metabolites [44]. Peroxidase, superoxide dismutase, glutathione reductase, and dehydroascorbate reductase activity—in addition to that of other enzymes related to antioxidant and free radical scavenging—decreased due to seed aging in many crops [45,46]. Glutathione reduces fatty acids and nucleic acids to form corresponding monohydroxyl alcohols under the action of GSTs. This reduction plays a key role in preventing the degradation of organic hydroperoxide into aldehyde derivatives of cytotoxin [44,47,48]. Glutathione in rice and maize interacts physiologically and genetically with the bZIP transcription factors of the TGA family to control developmental and stress-related processes [49]. The considerable evidence above suggests that *GRMZM2G343519* may be involved in mediating differences in artificial aging that modifies seedling establishment capability.

*GRMZM2G074309*, *GRMZM2G117319*, and *GRMZM2G465812* correspond to terpene synthase 5 (*TPS5*), terpene synthase 4 (*TPS4*), and terpene synthase 9 (*TPS9*), respectively. Terpenes are the largest group of plant secondary metabolites, showing great diversity in carbon skeletons and functional groups. A single plant can contain up to 100 different terpenes [50,51]. Terpenoids act as photosynthetic pigments (carotenoids), regulators of growth and development (such as gibberellins, key factors for seed germination, abscisic acid, strigolactone, brassino steroids, and cytokinins), and play an important role in protein glycosylation [52]. Toxic substances accumulated during seed aging indicate that seeds are under abiotic stress. Many terpenoids are involved in the protection of plants from abiotic stresses [53]. *TPS21* and *TPS11* react more directly to jasmonic acid (JA). Their promoters bind directly to the bHLH transcription factor *MYC2*, which is a central regulator of the JA signaling pathway in developmental and stress responses [54,55,56]. In addition, the overexpression of *TPS11* in maize, and the terpene synthase gene *OsTPS19* in rice, could enhance resistance to fungal pathogens [57,58]. Thus, these three genes may be related to seed vigor after aging.

In conclusion, SLAF-seq is a very efficient method for the construction of high-density genetic maps for crops, and the four candidate genes identified through SLAF-seq provide the theoretical basis and support for the cultivation of high-vigor varieties of sweet corn.

## 5. Conclusions

In this study, a BC_4_F_3_ population was created and a high-density genetic linkage map constructed with a mean genetic distance of 0.62 cM between adjacent markers using SLAF. A total of 57.06 Gb raw data were generated, including 228.26 M reads. The remaining length after pretreatment was 100 bp. The final genetic map consisted of 3876 markers on chromosome 10, with a length of 2413.25 cM. A total of 18 QTLs of four germination traits were detected after exposure to artificial aging conditions, based on this high-density genetic map. Among them, *qGR10* was highly reliable and will be investigated in detail in a future study. Within this interval, combining gene annotation, we found four promising candidate genes (*GRMZM2G343519*, *GRMZM2G074309*, *GRMZM2G117319*, and *GRMZM2G465812*) which may be related to seed vigor after aging. These results are helpful for deepening our understanding of the genetic mechanisms of artificial aging in establishing seedlings.

## Figures and Tables

**Table 1:** Summary of sequencing data of samples.

Sample	Total Reads	GC Percentage (%) ^1^	Q30 Percentage (%) ^2^	Marker Number	Total Depth	Average Depth
M03	12,759,218	45.42	85.74	135,956	4,995,576	36.74
M08	12,276,986	45.58	85.02	90,506	4,305,095	47.57
Offspring	1,370,055	45.15	86.39	98,023	536,053	5.47

^1^ Percentage of guanine (G) and cytosine (C) bases in total bases. ^2^ Percentage of bases with sequencing quality value greater than or equal to 30.

**Table 2:** Basic details of the genetic map.

Chr ^1^	Marker Number	Total cM ^2^	Average cM ^3^	Max Gap ^4^	Gap < 5 cM
Chr1	475	329.92	0.69	14.2	95.14%
Chr2	353	230.88	0.65	14.0	90.86%
Chr3	464	294.87	0.64	12.2	92.74%
Chr4	433	288.80	0.67	8.98	97.24%
Chr5	405	257.25	0.64	15.28	98.61%
Chr6	343	214.57	0.63	9.33	99.26%
Chr7	381	237.86	0.62	7.03	98.49%
Chr8	325	207.57	0.64	6.32	97.31%
Chr9	179	114.32	0.64	4.97	96.84%
Chr10	518	237.22	0.46	8.1	97.94%
Total	3876	2413.25	0.62	15.28	96.44%

^1^ Chr: chromosome. ^2^ Total genetic distance of markers on a chromosome. ^3^ Average genetic distance of markers on a chromosome. ^4^ Maximum gap on chromosome. The smaller the maximum gap, the more uniform the genetic map.

**Table 3:** Phenotypic performance of traits related to seed vigor in the BC_4_F_3_ seeds after artificial aging.

Season	Trait	Parents (Mean ± SD)		BC_4_F_3_ Population		Skewness	Kurtosis	CV (%)	h^2^
		M03	M08	Range	Mean ± SD				
2017S	GP	0.82 ± 0.23	0.36 ± 0.18	0.04–1.00	0.47 ± 0.19	−0.06	−0.42	41.02	0.72
	GR	0.92 ± 0.13	0.55 ± 0.11	0.20–1.00	0.79 ± 0.14	−1.25	2.33	17.81	0.64
	GI	3.86 ± 0.11	1.32 ± 0.54	0.52–4.24	2.78 ± 0.62	−0.65	0.84	22.32	0.66
	VI	26.47 ± 5.39	11.44 ± 4.76	0.24–32.41	13.73 ± 6.26	0.48	0.39	45.64	0.73
2017A	GP	0.79 ± 0.16	0.36 ± 0.17	0.00–0.88	0.42 ± 0.21	0.01	−0.80	49.20	0.73
	GR	0.85 ± 0.20	0.53 ± 0.15	0.16–1.00	0.77 ± 0.14	−1.28	3.63	18.42	075
	GI	3.43 ± 0.82	1.37 ± 0.56	0.40–4.04	2.63 ± 0.65	−0.56	0.94	24.61	0.74
	VI	24.26 ± 5.27	8.92 ± 3.26	0.19–30.76	11.79 ± 5.76	0.58	0.49	18.91	0.67

CV: coefficient of variation; GP: germination potential; GR: germination rate; GI: germination index; VI: vigor index; h^2^ refers to broad sense heritability.

**Table 4:** QTLs detected for seed vigor under artificial aging treatments in the BC_4_F_3_ population.

Season	Trait	QTL	Chr ^1^	QTL Region (cM)	Marker Interval	LOD ^2^	ADD ^3^	R^2^ (%) ^4^
2017S	GP	*qGP6*	6	38.800–38.810	Marker1435–Marker1452	3.01	0.15	1.69
		*qGP7-1*	7	0.000–0.040	Marker2623–Marker2627	2.40	−0.09	0.62
	GR	*qGR10*	10	0.000–1.400	Marker393–Marker398	3.24	−0.20	5.53
		*qGR6*	6	113.746–113.786	Marker1652–Marker1656	3.55	−0.13	2.38
	GI	*qGI7-1*	7	0.000–0.040	Marker2623–Marker2627	4.24	−0.46	1.49
	VI	*qVI7-1*	7	0.000–0.040	Marker2623–Marker2627	3.90	−4.04	1.15
2017A	GR	*qGR10*	10	0.000–1.400	Marker393–Marker398	5.85	−0.31	13.14
	GI	*qGI10*	10	0.000–1.400	Marker393–Marker398	3.96	−1.12	8.07
	VI	*qVI1*	1	282.283–282.343	Marker8370–Marker8371	2.16	6.08	3.03
		*qVI3-1*	3	120.160–122.540	Marker4631–Marker4636	2.28	−4.57	1.71
		*qVI3-2*	3	220.090–221.731	Marker4866–Marker4867	2.50	−2.48	0.50
		*qVI3-3*	3	36.250–36.280	Marker4245–Marker4248	2.06	3.04	0.76
		*qVI3-4*	3	81.440–81.470	Marker4749–Marker4752	2.27	−4.63	1.76
		*qVI5-1*	5	243.962–243.972	Marker4126–Marker4127	2.05	6.01	2.96
		*qVI9*	9	56.586–56.596	Marker1052–Marker1053	2.17	6.06	3.02
		*qVI4*	4	206.290–207.931	Marker6830–Marker6834	2.53	4.93	2.02
		*qVI5-2*	5	90.936–90.946	Marker3585–Marker3588	2.95	5.62	2.63
		*qVI7-2*	7	3.395–3.405	Marker2628–Marker2631	3.00	−2.73	0.62

^1^ Chr: chromosome; ^2^ LOD: logarithm of odds score; ^3^ Positive and negative values indicate additive effects by the alleles of M03 and M08, respectively; ^4^ R^2^ phenotypic variance explained by an individual QTL.

## Supplementary Materials

The following are available online at: https://www.mdpi.com/2073-4425/11/1/37/s1, Table S1: Chromosome distribution statistics of SLAF labels and polymorphic SLAF labels, Table S2: Coefficient of correlation between genetic maps and genomes among linkage groups, Table S3: Gene annotation of the 25 genes with the interval of *qGR10*.
