# Supplementary material for: Construction of High-Density Genetic Map and Identification of QTLs Associated with Seed Vigor after Exposure to Artificial Aging Conditions in Sweet Corn Using SLAF-seq
Source: Genes (Basel). 2019 Dec 28;11(1):37. doi: 10.3390/genes11010037 (PMC7016829; doi:10.3390/genes11010037)
Supplement: Supplementary file 1 [file genes-11-00037-s001.zip › Table S1.docx]

**Table S1.** Chromosome distribution statistics of SLAF labels and polymorphic SLAF labels

| **LG ID^a^** | **Total SLAF lables** | **Polymorphic SLAF labels** |
| --- | --- | --- |
| Chr1 | 23349 | 3972 |
| Chr2 | 18165 | 3828 |
| Chr3 | 18289 | 3470 |
| Chr4 | 20679 | 3841 |
| Chr5 | 16946 | 2957 |
| Chr6 | 12901 | 2296 |
| Chr7 | 13543 | 2640 |
| Chr8 | 14357 | 3452 |
| Chr9 | 13348 | 3033 |
| Chr10 | 11652 | 1997 |
| Total | 163229 | 31486 |
