# Supplementary material for: Construction of High-Density Genetic Map and Identification of QTLs Associated with Seed Vigor after Exposure to Artificial Aging Conditions in Sweet Corn Using SLAF-seq
Source: Genes (Basel). 2019 Dec 28;11(1):37. doi: 10.3390/genes11010037 (PMC7016829; doi:10.3390/genes11010037)
Supplement: Supplementary file 1 [file genes-11-00037-s001.zip › Table S2.docx]

**Table S2.** Coefficient of correlation between genetic maps and genomes among linkage groups

| LG ID^1^ | Spearman |
| --- | --- |
| Chr1 | 0.82 |
| Chr2 | 0.62 |
| Chr3 | 0.93 |
| Chr4 | 0.87 |
| Chr5 | 0.92 |
| Chr6 | 0.73 |
| Chr7 | 0.71 |
| Chr8 | 0.93 |
| Chr9 | 0.99 |
| Chr10 | 0.63 |

^1^Chr chromosome
