# Supplementary material for: Construction of High-Density Genetic Map and Identification of QTLs Associated with Seed Vigor after Exposure to Artificial Aging Conditions in Sweet Corn Using SLAF-seq
Source: Genes (Basel). 2019 Dec 28;11(1):37. doi: 10.3390/genes11010037 (PMC7016829; doi:10.3390/genes11010037)
Supplement: Supplementary file 1 [file genes-11-00037-s001.zip › Table S3.docx]

**Table S3.** Gene annotation of the 25 genes with the interval of *qGR10*

| **GeneID** | **Gene annotation** |
| --- | --- |
| AC203341.3_FG | low_confidence |
| AC203341.3_FG | *ZEAMMB73_148384* |
| AC215701.3_FG | low_confidence |
| AC232320.1_FG | low_confidence |
| GRMZM2G002976 | uncharacterized LOC103642531 |
| GRMZM2G022506 | uncharacterized LOC100191215 |
| GRMZM2G048656 | low_confidence |
| GRMZM2G062807 | WD40-like domain containing protein putative expressed |
| GRMZM2G074309 | [tps5 - terpene synthase5](https://www.maizegdb.org/gene_center/gene/tps5) |
| GRMZM2G117319 | [tps4 - terpene synthase4](https://www.maizegdb.org/gene_center/gene/tps4) |
| GRMZM2G127602 | low_confidence |
| GRMZM2G133512 | DNA-directed RNA polymerases IV and V subunit 2 |
| GRMZM2G148316 | UDP-glycosyltransferase 85A5 |
| GRMZM2G154455 | low_confidence |
| GRMZM2G176355 | orphan gene ZEAMMB73_353506 |
| GRMZM2G343519 | Glutaredoxin family protein |
| GRMZM2G380765 | ZEAMMB73_033414 |
| GRMZM2G380776 | *ZEAMMB73_828935* |
| GRMZM2G384763 | hypothetical protein ZEAMMB73_Zm00001d024482 |
| GRMZM2G407495 | [hypothetical protein ZEAMMB73_Zm00001d024471](https://www.ncbi.nlm.nih.gov/protein/AQK41271.1) |
| GRMZM2G465812 | [tps9 - terpene synthase9](https://www.maizegdb.org/gene_center/gene/tps9) |
| GRMZM2G700927 | *ZEAMMB73_471428* |
| GRMZM5G848529 | low_confidence |
| GRMZM5G859928 | low_confidence |
| GRMZM5G863023 | ZEAMMB73_669610 |
